# Asymmetric dimethylarginine (ADMA), symmetric dimethylarginine (SDMA) and homoarginine (hArg): the ADMA, SDMA and hArg paradoxes

**DOI:** 10.1186/s12933-017-0656-x

**Published:** 2018-01-04

**Authors:** Dimitrios Tsikas, Alexander Bollenbach, Erik Hanff, Arslan Arinc Kayacelebi

**Affiliations:** 1 Institute of Toxicology, Core Unit Proteomics, Hannover Medical School, 30623 Hannover, Germany; 2 Institute of Toxicology, Core Unit Proteomics, Hannover Medical School, Carl-Neuberg-Strasse 1, 30625 Hannover, Germany

**Keywords:** l-Arginine, Cardiovascular disease, Diabetes, l-Homoarginine, Inhibition, Methylated l-arginine, Nitric oxide, Nitric oxide synthase, Risk factor, Risk marker

## Abstract

*N*^G^-Methylation of l-arginine (Arg) residues in certain proteins by protein arginine methyltransferases and subsequent proteolysis yields *N*^G^-monomethyl-l-arginine (MMA), *N*^G^,*N*^G^-dimethyl-l-arginine (asymmetric dimethylarginine, ADMA) and *N*^G^,*N*′^G^-dimethyl-l-arginine (symmetric dimethylarginine, SDMA). Biological MMA, ADMA and SDMA occur as free acids in the nM-range and as residues of proteins of largely unknown quantity. Arginine:glycine amidinotransferase (AGAT) catalyzes the synthesis of L-homoarginine (hArg) from free Arg and l-lysine. Biological hArg is considered to occur exclusively as free acid in the lower µM-range. Nitric oxide synthase (NOS) catalyzes the conversion of Arg (high affinity) and hArg (low affinity) to nitric oxide (NO) which is a pleiotropic signaling molecule. MMA, ADMA and SDMA are inhibitors (MMA > ADMA ≫ SDMA) of NOS activity. Slightly elevated ADMA and SDMA concentrations and slightly reduced hArg concentrations in the circulation are associated with many diseases including diabetes mellitus. Yet, this is paradox: (1) free ADMA and SDMA are weak inhibitors of endothelial NOS (eNOS) which is primarily responsible for NO-related effects in the cardiovascular system, with free hArg being a poor substrate for eNOS; (2) free ADMA, SDMA and hArg are not associated with oxidative stress which is considered to induce NO-related endothelial dysfunction. This ADMA/SDMA/hArg paradox may be solved by the assumption that not the free acids but their precursor proteins exert biological effects in the vasculature, with hArg antagonizing the effects of *N*^G^-methylated proteins.

## Background

The nitric oxide synthase (NOS) family comprises the endothelial NOS (eNOS), the neuronal NOS (nNOS) and the inducible NOS (iNOS). These NOS isoforms catalyze the conversion of l-arginine (l-Arg) and l-homoarginine (l-hArg) to nitric oxide (NO), one of the most potent physiological vasodilators and inhibitors of platelet aggregation. NO and other endothelium-derived substances including prostacyclin (vasodilator and platelet function inhibitor) and endothelin (vasoconstrictor) are considered to play major roles in the cardiovascular system. Altered homeostasis of endothelium-derived NO due to dysfunctional endothelium is generally assumed to result in cardiovascular disease. The NO metabolite nitrite in the circulation is a surrogate of endothelium-derived short-lived analytically inaccessible NO.

Certain proteins are *N*^G^-methylated by protein l-arginine methyltransferases (PRMTs). Their proteolysis releases the free acids of *N*^G^-monomethyl-l-arginine (MMA), *N*^G^,*N*^G^-dimethyl-l-arginine (asymmetric dimethylarginine, ADMA), and *N*^G^,*N*′^G^-dimethyl-l-arginine (symmetric dimethylarginine, SDMA). The NOS-catalyzed formation of NO from l-Arg is inhibited by the free forms of MMA, ADMA and SDMA. The concentration of the latter in the circulation of healthy humans is of the order of 100, 400 and 400 nM, respectively. Concentration and functions of *N*^G^-methylated l-Arg proteins, i.e., the precursors of MMA, ADMA and SDMA, are largely unknown. Given the relatively low MMA concentration, the scientific interest was originally focused on ADMA and SDMA. Compared to healthy subjects, the concentrations of circulating ADMA and SDMA are higher in many cardiovascular and renal diseases including diabetes mellitus. Free ADMA was first identified as a cardiovascular risk factor. Free SDMA was only recently identified as a cardiovascular risk factor, with some studies revealing SDMA even as a more significant cardiovascular and renal risk factor than free ADMA and MMA [1]. In this context, it is notable that ADMA plasma levels did not differ among patients with dissimilar glomerular filtration rate values [2]. The observation of the higher cardiorenal significance of SDMA was highly unexpected in the scientific community because free SDMA was generally considered not to be an NOS inhibitor. To overcome this contradiction, an alternative mechanism has been proposed, namely the potential of free SDMA and free ADMA to induce oxidative stress which is generally assumed to be a major contributor to cardiovascular disease. Unlike ADMA and SDMA, low circulating and urinary concentrations of free l-hArg were found to be associated with elevated cardiovascular risk, morbidity and mortality. This finding was surprising because l-hArg was considered a non-physiological and non-proteinogenic amino acid until recently. Thus far, there is no convincing explanation that just reduced concentrations of free l-hArg in the circulation are associated with cardiovascular risk.

A closer examination reveals that neither the inhibitory action of free ADMA and SDMA on eNOS nor the oxidative potential of free ADMA, SDMA and L-hArg, not to mention the negligible contribution of l-hArg to NO, can explain the statistically observed associations of free ADMA, SDMA and l-hArg with cardiovascular disease. This examination and our arguments against l-Arg/NOS-based effects of ADMA, SDMA and hArg in the cardiovascular system are outlined and discussed below in detail.

## Discussion

### MMA, ADMA and SDMA as inhibitors of, and hArg as substrate for NO synthesis

In 1992, Vallance et al. [3] reported that ADMA and MMA, but not SDMA, inhibited iNOS activity in J774 macrophage cytosol (by 18% at 5 µM ADMA), and that ADMA (EC_50_, 26 µM) contracted endothelium-intact rat aortic rings. In the same study, ADMA infusion (25 µmol/kg/h) raised systolic blood pressure by nearly 15% at a plasma concentration of about 10 µM in anaesthetized Guinea pigs, whereas ADMA infusion (8 µmol for 5 min into the brachial-artery) decreased forearm blood-flow by 28% in healthy humans [3]. The authors stated in their article that free ADMA and MMA, but not free SDMA, inhibited NO synthesis in vitro and in vivo. Since then, the report by Vallance et al. [3] received much attention, is a much-quoted and landmark paper concerning the particular importance of ADMA, but not of SDMA, as an endogenous inhibitor of NO synthesis [3]. The focus on ADMA given subsequently by the scientific community is a plausible explanation for numerous studies that eventually revealed free ADMA as a cardiovascular risk factor in humans, while free SDMA was left entirely in the shadow of ADMA. Thus, it took many years until SDMA has attracted particular attention, beyond its importance as a uremic toxin, and has also emerged as a cardiovascular risk factor, in some studies hand-in-hand with ADMA [1, 4, 5]. This is a big surprise when considering that free SDMA has been and still is generally considered not to be an inhibitor of NO synthesis. One may therefore ask the question, whether the scientific community has uncritically adopted previous observations [3] and ignored or overlooked important information.

It is indeed very surprising, that, despite the substantial interest in ADMA and its undoubtful importance in the cardiovascular system, only very scare information is available about the inhibitory potency of free ADMA towards all known NOS isoforms, i.e., eNOS, nNOS, iNOS, and the underlying mechanisms of the inhibition of NOS activity [6]. A likely reason could be the generalization of the results reported by Vallance et al. [3] with respect to the rather very weak inhibitory action of free ADMA and their uncritical extension to eNOS, and the almost complete neglect of free SDMA in the subsequent years.

By using a recombinant nNOS, we found that not only free ADMA, MMA, *N*^G^-nitro-l-arginine (NNA) and *N*^G^-nitro-l-arginine methyl ester (l-NAME), but also free SDMA inhibit nNOS activity (discussed in Ref. [6]). At a molar basis (1 µM), the order of the inhibitory potency towards nNOS activity was approximately 6:5:4:1:1 for NNA:ADMA:NMA:L-NAME:SDMA. Thus, compared to ADMA, SDMA is a considerably less potent inhibitor of nNOS activity. Free ADMA is a potent (IC_50_, 1.5 µM) noncompetitive inhibitor of recombinant nNOS activity, but a very weak (IC_50_, 12 µM) competitive inhibitor of eNOS activity [6]. To the best of our knowledge, there are no reported data on the inhibitory potency of SDMA towards eNOS activity. Presumably, free SDMA is a weak inhibitor of eNOS, most likely weaker than ADMA. The quite weak inhibitory potency of ADMA and presumably of SDMA towards eNOS activity is not compatible with the key importance attributed to these methylated L-Arg derivatives in the human circulation.

Free l-hArg has been demonstrated to be converted to NO by recombinant eNOS, nNOS and iNOS; yet, l-hArg was found to have a much lower affinity to these enzymes when compared with l-Arg [7]. Because of this and the very low circulating and tissue l-hArg concentrations, notably in relation to free l-Arg, free l-hArg cannot be considered as an important substrate and much less as an inhibitor of eNOS.

### Are ADMA, SDMA and hArg inducers of oxidative stress?

ADMA is generally associated with elevated oxidative stress, although no solid evidence has been provided thus far. In patients suffering from type 2 diabetes mellitus (T2DM), no association between free ADMA and oxidative stress was observed [8]. Treatment of the T2DM patients with an angiotensin II receptor antagonist did reduce the oxidative stress level, but did not alter the plasma ADMA concentration [8]. With regard to an association of SDMA with oxidative stress, the body of evidence is even much thinner. To the best of our knowledge, there is only in vitro indication that free SDMA and ADMA may very weakly increase “oxidative burst” in *N*-formyl-methionine-leucine-phenylalanine (fMLP)-stimulated monocytes [9].

In women with normal (Group 1) or impaired (Group 2, normal outcome; Group 3, intrauterine growth restriction; Group 4, preeclampsia) placental function [10], a moderate correlation (*r* = 0.58, *P* = 0.0009) was found between ADMA and SDMA plasma concentrations only in the healthy pregnant women (Table 1). This correlation is of the same order of magnitude (*r* = 0.45, *P* < 0.001) reported by Zobel et al. [5] for T2DM patients. In our study, neither SDMA nor ADMA plasma concentration correlated with the plasma concentration of the oxidative stress biomarker *cis*-epoxyoleic acid (*cis*-EpOA) (Table 1) [10]. This finding suggests that neither SDMA nor ADMA are associated with oxidative stress in normal or abnormal pregnancy.

**Table 1:** Correlation (by Spearman) between the plasma concentrations of ADMA, SDMA and *cis*-EpOA in women with normal (Group 1) or impaired placental function (Groups 2, 3, 4)

	ADMA vs SDMA	ADMA vs *cis*-EpOA	SDMA vs *cis*-EpOA
Group 1	*r* = *0.58*, *P* = *0.0009*, *n* = *30*	*r* = 0.05, *P* = 0.79, *n* = 30	*r* = − 0.07, *P* = 0.72, *n* = 29
Group 2	*r* = 0.05, *P* = 0.85, *n* = 16	*r* = 0.42, *P* = 0.11, *n* = 16	*r* = − 0.42, *P* = 0.10, *n* = 16
Group 3	*r* = 0.29, *P* = 0.33, *n* = 14	*r* = − 0.20, *P* = 0.51, *n* = 13	*r* = 0.10, *P* = 0.75, *n* = 13
Group 4	*r* = 0.26, *P* = 0.47, *n* = 10	*r* = 0.31, *P* = 0.39, *n* = 10	*r* = − 0.18, *P* = 0.63, *n* = 10
Groups 2,3,4	*r* = 0.15, *P* = 0.35, *n* = 40	*r* = 0.08, *P* = 0.64, *n* = 39	*r* = − 0.21, *P* = 0.21, *n* = 39

In summary, there is no convincing evidence of an appreciable oxidative potential of free ADMA, SDMA and l-hArg

In vitro in HUVEC and in vivo in the rat (i.p. injection up to 400 mg/kg bodyweight), exogenous free l-hArg was found not to induce oxidative stress [11].

Paraoxonases (PON) possess several biological functions, yet their primary role is still speculative. PON-1 is thought to be associated with lipid peroxidation. In patients suffering from T2DM and dyslipidemia, 12-weeks treatment with rosuvastatin was reported to increase serum PON-1 activity and to lower plasma ADMA concentration [12]. Yet, a correlation between PON-1 and ADMA seems not to have occurred in that study at baseline or after rosuvastatin treatment.

### *N*^G^-Methylated and guanidinated proteins versus free ADMA, SDMA and hArg

Free l-hArg is synthesized from free l-Arg and l-lysine by l-arginine:glycine amidinotransferase (AGAT), with l-ornithine being the second reaction product. AGAT also catalyzes the reaction of l-Arg and glycine to form guanidinoacetate (GAA), the precursor of the energy-related creatine. l-hArg is considered to be a non-proteinogenic amino acid. Thus far, there are no reports that proteins comprise l-hArg residues due to physiological post-translational modifications. Yet, it has been demonstrated that synthetic peptides that contain alkylated hArg or Arg residues in position 6 possess strong biological activity, presumably via interaction of the positively charged hArg moiety with the negatively charge phosphate group of the phospholipid cell membrane [13]. The most feasible possibility for the formation of l-hArg-containing proteins could be guanidination of the terminal amine group of l-Lys residues, analogous to the widely occurring and abundant *N*^ε^-methylation.

In accordance with the prevailing doctrine, free MMA, ADMA and SDMA are released by regular proteolysis of *N*^G^-methylated proteins. Identity, concentration and potential roles of the majority of those proteins in the renal, cardio- and cerebrovascular systems are largely unknown. It is worth mentioning that *N*^G^-methylated proteins may play a role in insulin signaling in L6 skeletal muscle cells [14]. Should *N*^G^-methylated proteins and guanidinated proteins possess intrinsic biological activity, there arise several questions. Do *N*^G^-methylated and guanidinated proteins act antagonistically? Are free ADMA, SDMA and l-hArg risk markers or risk factors in human disease such as diabetes mellitus? Does the concentration of free ADMA, SDMA and l-hArg in the circulation reflect the tissue concentration of *N*^G^-methylated and guanidinated proteins? These issues warrant deeper investigations.

## Conclusions

Free MMA, ADMA and SDMA, are endogenous inhibitors of NOS activity with differences in type, efficacy and potency of inhibition. eNOS and oxidative stress are considered the most important factors in the cardiovascular system. The weak inhibitory potency of free ADMA and SDMA towards eNOS, the low affinity of free L-hArg to eNOS, and the lacking oxidant potential of free ADMA, SDMA and l-hArg argue against an important role of these l-Arg relatives in NO-related endothelial dysfunction. The emergence of free ADMA, SDMA and l-hArg as cardiovascular risk factors appears paradoxical. The connection of ADMA, SDMA and l-hArg with NO is plausible and cannot be entirely dismissed. However, there is no convincing evidence that the hazard risk emerging from or is associated with free ADMA, SDMA and l-hArg is rooted in the weak inhibition of NO synthesis by slightly elevated concentrations of free ADMA and SDMA, and/or in the negligibly low contribution of free l-hArg as eNOS substrate by slightly decreased concentrations of l-hArg observed in many diseases. A solution of the free ADMA, SDMA and l-hArg paradoxes in the cardiovascular system could be antagonistic actions of *N*^G^-methylated and guanidinated proteins.

## Abbreviations

ADMA: asymmetric dimethylarginine
hArg: homoarginine
MMA: monomethylarginine
NO: nitric oxide
NOS: nitric oxide synthase
SDMA: symmetric dimethylarginine
T2DM: type 2 diabetes mellitus
